# Immune evaluation of granulocyte-macrophage colony stimulating factor loaded hierarchically 3D nanofiber scaffolds in a humanized mice model

**DOI:** 10.3389/fbioe.2023.1159068

**Published:** 2023-03-24

**Authors:** Rui Chen, Yujie Li, Yangyang Zhuang, Yiming Zhang, Hailong Wu, Tao Lin, Shixuan Chen

**Affiliations:** ^1^ Jiangsu Key Laboratory of Marine Bioresources and Environment, Jiangsu Ocean University, Lianyungang, China; ^2^ Zhejiang Engineering Research Center for Tissue Repair Materials, Wenzhou Institute, University of Chinese Academy of Sciences, Wenzhou, China; ^3^ Department of Plastic, Reconstructive and Cosmetic Surgery, Xinqiao Hospital, Army Medical University, Chongqing, China; ^4^ Department of Intensive Care Unit, The First Affiliated Hospital of Wenzhou Medical University, Wenzhou, China; ^5^ Oujiang Laboratory (Zhejiang Lab for Regenerative Medicine, Vision and Brain Health), Wenzhou, China

**Keywords:** granulocyte-macrophage colony stimulating factor (GM-CSF), 3D radially aligned nanofiber scaffolds, cell migration, immune responses, tissue regeneration

## Abstract

**Background:** Immune evaluation of biomaterials for tissue regeneration is a critical preclinical evaluation. The current evaluation criterion (ISO 10993-1 or GB/T 16886) uses rodents to perform the immune evaluation. However, the immune system of rodents is different from humans, the obtained results may not be reliable, which could lead directly to the failure of clinical trials. Granulocyte-macrophage colony-stimulating factor (GM-CSF) shows a great potential application in tissue regeneration by regulating local immune responses. The presented work combines the advantages of GM-CSF (immunoregulation) and hierarchically 3D nanofiber scaffolds (tissue regeneration).

**Methods:** Firstly, we fabricated GM-CSF loaded 3D radially aligned nanofiber scaffolds, and then subcutaneous implantation was performed in humanized mice. The whole scaffold and surrounding tissue were harvested at each indicated time point. Finally, the cell infiltration and local immune responses were detected by histological observations, including H&E and Masson staining and immunochemistry.

**Results:** We found significant cell migration and extracellular matrix deposition within the 3D radially aligned nanofiber scaffold after subcutaneous implantation. The locally released GM-CSF could accelerate the expression of human dendritic cells (CD11c) only 3 days after subcutaneous implantation. Moreover, higher expression of human cytotoxic T cells (CD3^+^/CD8^+^), M1 macrophages (CD68/CCR7) was detected within GM-CSF loaded radially aligned nanofiber scaffolds and their surrounding tissues.

**Conclusions:** The 3D radially aligned scaffold can accelerate cell migration from surrounding tissues to regenerate the wound area. And the locally released GM-CSF enhances dendritic cell recruitment and activation of cytotoxic T cells and M1 macrophages. Taken together, the GM-CSF loaded 3D radially aligned nanofiber scaffolds have a promising potential for achieving tissue regeneration.

## Introduction

Granulocyte-macrophage colony stimulating factor (GM-CSF) is a glycoprotein-based growth factor (Fleetwood et al., 2005). It can regulate the immune functions of the human body, including enhancing the body’s resistance and regulating the functions of repair cells and inflammatory cells (Lim et al., 2013; Hamilton, 2020). In the field of wound healing, it has been reported that GM-CSF can prevent wound infection and healing by modulating local immune activity (Zhang et al., 2010). The immunoregulatory function of GM-CSF is mainly reflected in the recruitment and differentiation of dendritic cells and T lymphocytes (Shi et al., 2006; Chuang et al., 2021), and T lymphocytes play a significant role. T cells (standard marker: CD3^+^) are a fundamental immune system component. It is mainly divided into helper T cells (CD3^+^/CD4^+^) and cytotoxic T cells (CD3^+^/CD8^+^). The ratio of helper T cells/cytotoxic T cells is able to determine the status of local immune responses (Wang et al., 2017b; Li et al., 2020). When the expression of cytotoxic T cells (CD3^+^/CD8^+^) is dominant, the local immune responses will be towards anti-infection and removing necrotic tissue (Jannie et al., 2018; Chen Q. et al., 2019; Flippe et al., 2019). Thus, the GM-CSF has the potential to be used for immunoregulation. We hypothesize that loading GM-CSF to an ideal biomaterial scaffold and achieve tissue regeneration. Our previous study has focused on making a porous 3D nanofiber scaffold to mimic the structure of a natural extracellular matrix (Chen et al., 2018; Chen et al., 2019c; Chen et al., 2020a; Chen et al., 2021). It shows attractive characteristics, including good biocompatibility, big pore size and high porosity, high specific surface area, controlled fiber orientation, and ease of incorporation of drugs or growth factors (Greiner and Wendorff, 2007). It can accelerate cell penetration, ECM deposition, and angiogenesis (Sun et al., 2014; Chen et al., 2019c; Chen et al., 2020b). Among our developed 3D nanofiber scaffolds, the 3D radially aligned nanofiber scaffold has unique advantages, which can recruit cells from all directions (Chen et al., 2020a; Chen et al., 2021). In this study, we prepared GM-CSF loaded 3D radially aligned nanofiber scaffolds by electrospinning in combination with the subcritical CO_2_ fluid expansion technique.

Immune evaluation of biomaterials for tissue regeneration is a critical preclinical evaluation (Franz et al., 2011; Chung et al., 2017; Sadtler et al., 2019; Abaricia et al., 2021). The current gold standard evaluation criterion (ISO 10993-1 or GB/T 16886) uses rodents to perform the immune evaluation (Wang et al., 2017b; Li et al., 2020). However, the immune system of rodents is different from humans, the obtained results may not be reliable, which could lead directly to the failure of clinical trials. To reflect the accurate immune response of the GM-CSF loaded 3D radially nanofiber scaffolds in the human body, we used humanized mice produced by injecting CD34^+^ hematopoietic stem cells (Chen et al., 2019b). It can maintain a robust, long-term multi-lineage engraftment of human immune cell populations, resulting in the obtained results of immune responses are more credible, which will help to improve the success probability of subsequent clinical trials.

In this study, the humanized mouse was used to evaluate immune responses of granulocyte-macrophage colony stimulating factor loaded hierarchically 3D nanofiber scaffolds. For one thing, we will explore cell penetration and granulation formation within the scaffold after subcutaneous implantation. For another, we will also detect the local immune cell infiltration (e.g., dendritic cells, helper T cells, and cytotoxic T cells).

We found the locally released GM-CSF could accelerate the expression of human dendritic cells (CD11c) only 3 days after subcutaneous implantation. Moreover, higher expression of human cytotoxic T cells (CD3+/CD8+), M1 macrophages (CD68/CCR7) was detected within GM-CSF loaded radially aligned nanofiber scaffolds and their surrounding tissues. Suggesting the GM-CSF loaded 3D radially aligned nanofiber scaffolds have a promising potential for immune regulation during tissue regeneration.

## Materials and methods

### Materials

PCL (Mw = 80 kDa) was purchased from Sigma-Aldrich (St. Louis, MO, United States). Dichloromethane (DCM) and N, N-dimethylformamide (DMF) were purchased from BDH Chemicals (Dawsonville, GA, United States). DAPI was obtained from Santa Cruz Biotechnology, Inc. (Dallas, TX, United States). Antibodies, including human CD8 (1:250), human CD4 (1:500), human CD3 (1:500), human CD11c (1:300) were ordered from Abcam (Cambridge, MA, United States). GM-CSF recombinant human protein and GM-CSF human ELISA kit were purchased from Invitrogen (Carlsbad, CA, United States).

### Preparation of GM-CSF loaded PCL nanofiber mat

The GM-CSF was dissolved in a 10% pluronic F-127 aqueous solution. Then, the GM-CSF containing pluronic F-127 solution and 10% PCL solution were fed to the inner (0.06 mL/h) and outer nozzles (0.6 mL/h) of co-axial spinnerets during co-axial electrospinning. A rotating drum was used to collect the core-sheath nanofibers. A 1 mm-thick aligned GM-CSF loaded PCL nanofiber mat was collected (Chen et al., 2017).

### Fabrication of GM-CSF loaded expanded radially aligned nanofiber scaffold

PCL nanofiber mats were first to cut into 1 mm × 4 mm rectangles in liquid nitrogen to avoid deformation on the edges. The side of the nanofiber mat perpendicular to the direction of the nanofiber alignment was fixed by thermo-treatment (85°C for 1 s). Next, ∼1 g of dry ice and one piece of nanofiber mat was put into a 30 mL Oak Ridge centrifuge tube, and the tube was sealed. After the dry ice transformed into CO_2_ fluid, we quickly loosened the cap and removed the expanded nanofiber scaffold from the tube. This procedure was repeated until the desired expansion was reached. The expanded, radially aligned PCL nanofiber scaffolds with and without GM-CSF loading were sterilized with ethylene oxide gas before subcutaneous implantation (Jiang et al., 2018; Chen et al., 2019b).

### SEM observation

After expansion, Pt sputter coating was performed on the surface of GM-CSF loaded radially aligned nanofiber scaffolds. Then the morphology of GM-CSF loaded radially aligned nanofiber scaffolds was characterized by SEM (FEI, Quanta 200, Oregon, United States).

### GM-CSF release profile

One GM-CSF loaded expanded radially aligned nanofiber scaffold was immersed in 2 mL distilled water at 37°C. The supernatant was collected at each indicated time-point, and subsequently, 2 mL of fresh filtered water was added. The concentration of released GM-CSF at each time point was measured using an ELISA kit.

### Subcutaneous implantation

The humanized mice used in this study were created as in our previous study (Chen et al., 2019b). The humanized mice were anesthetized using 4% isoflurane in oxygen for approximately 2 min. Mice were placed on a heating pad to maintain their body temperature. An area of 4 × 4 cm^2^ on the back of each animal was shaved, and the povidone-iodine solution was applied three times on the exposed skin. Subcutaneous pockets were made (1 cm incisions) on both sides of the dorsum. The GM-CSF encapsulated expanded radially aligned nanofiber scaffold was directly inserted into a subcutaneous pocket with tweezers, and the skin incisions were closed with a stapler. Each mouse received two scaffolds. Two mice were considered the treatment group to investigate four implants for each group. Mice were euthanized with CO_2_ at 3, 7, 10, and 14 days post-implantation. Each explant with the surrounding tissue was gently dissected out of its subcutaneous pocket and then immersed in formalin for at least 3 days before histology analysis.

### Histological observations

Fixed samples were dehydrated in a graded series of ethanol (70%–100%), embedded in paraffin, and then sectioned (5 μm thick). Samples were stained with either hematoxylin, eosin (H&E), or Masson’s trichrome stain.

### Immunofluorescent staining

For immunohistochemical staining, slides were deparaffinized and rehydrated, followed by antigen retrieval in heated citrate buffer for 5 min (citrate buffer solution, pH 6.0 at 100°C). Non-specific antibody binding was prevented with a 5% BSA solution. The sections were incubated with primary antibodies overnight at 4°C. Then the corresponding secondary antibodies were added and incubated for 1 h at room temperature, followed by staining with DAPI for 5 min. We counted all CD11c, CD3^+^/CD4^+^, and CD3^+^/CD8^+^ positive cells in each 200 μm^2^ area, and five randomly selected areas were used to determine the average positive cells per mm^2^.

### Statistical analysis

Data are presented as the mean ± S.D., and statistical analysis was performed using SPSS 13.0 software (IBM Corporation, Armonk, NY, United States). Differences among groups were assessed using one-way ANOVA followed by *post hoc* tests. The values of *p* < 0.05 were considered statistically significant. The values of *p* < 0.01 were considered statistically highly significant.

## Result

**FIGURE 1:**
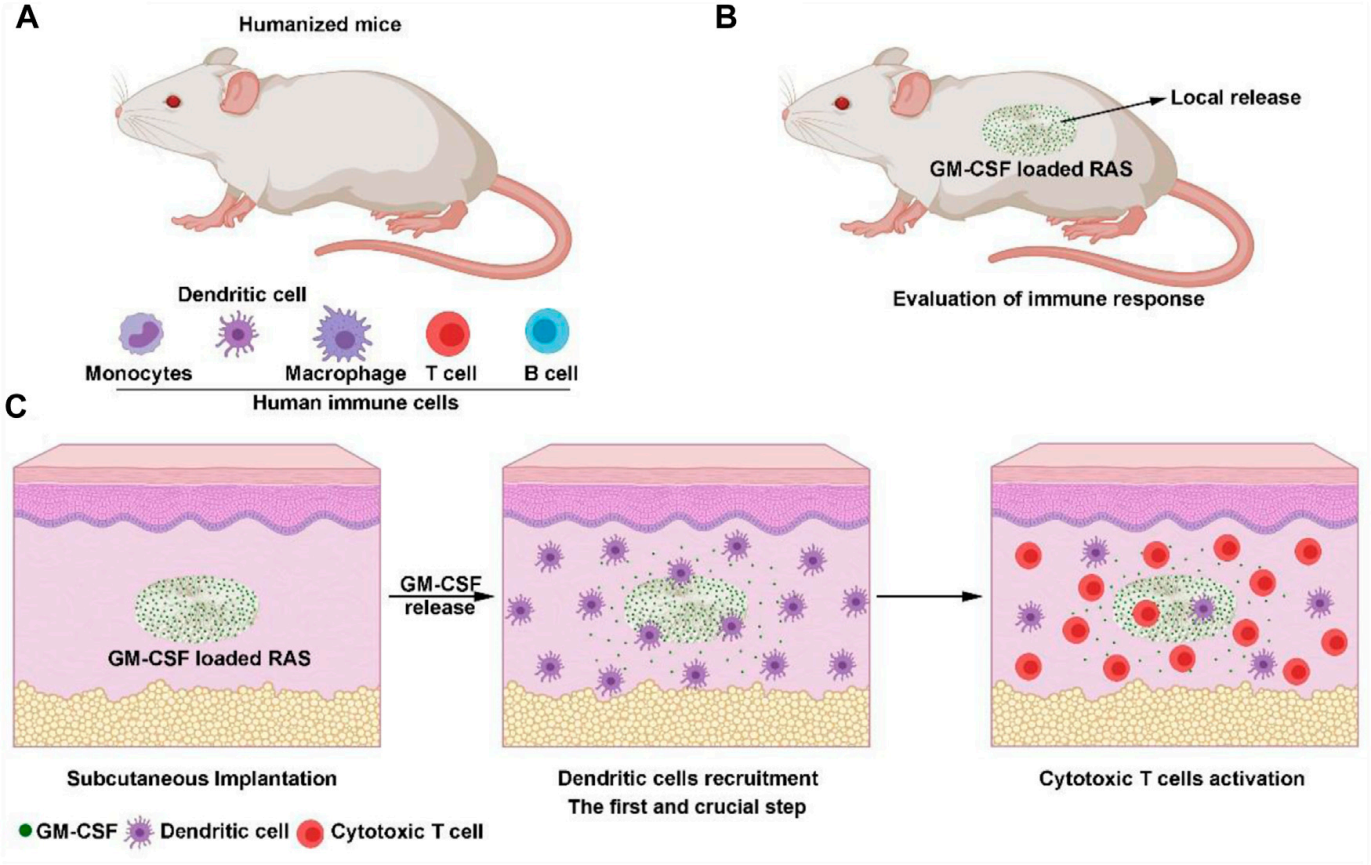
**(A)** Schematic illustrating humanized mice have a human immune system that contains human immune cells, including human monocytes, dendritic cells, macrophages, T cells, and B cells. **(B)** Schematic illustrating the evaluation of human immune responses to GM-CSF loaded, radially aligned PCL nanofiber scaffolds after subcutaneous implantation. **(C)** Schematic illustrating locally released GM-CSF promotes the recruitment of human dendritic cells and differentiation to cytotoxic T cells.

Despite those gratifying results, the *in vivo* evaluations of these studies were performed in rodents model, which provided a limited understanding of interactions between the human immune system and these therapies, resulting in the failure of a clinical trial, even inducing some unexpected side effects in patients. To address this limitation, humanized mice have been widely used for long-term *in vivo* studies in transplant rejection, cancer immunology, and infectious diseases because of their unique human immune system (Figure 1A) (Shultz et al., 2007; Legrand et al., 2009; Shultz et al., 2012). In recent years, it also began to be used for evaluating biocompatibility, human immune cell responses to biomaterials (Wang et al., 2017a; Wang et al., 2017b), and bioactive molecules loaded biomaterials (Chen et al., 2019b). In our previous studies, we fabricated 3D radially aligned nanofiber scaffolds with controlled porosity and tailored sizes, thicknesses, and shapes for the first time (Chen et al., 2019b; Chen et al., 2019c; Chen et al., 2020b). In the presented study, we first explored the human immune response to GM-CSF loaded radially aligned scaffolds (Figure 1B). We are trying to discover whether the radially aligned scaffolds could accelerate cell migration from surrounding tissue, and whether the local released GM-CSF could enhance the recruitment of dendritic cells and the activation of cytotoxic T cells (Figure 1C).

### Characterization of 3D radially aligned nanofiber scaffolds and release kinetics

**FIGURE 2:**
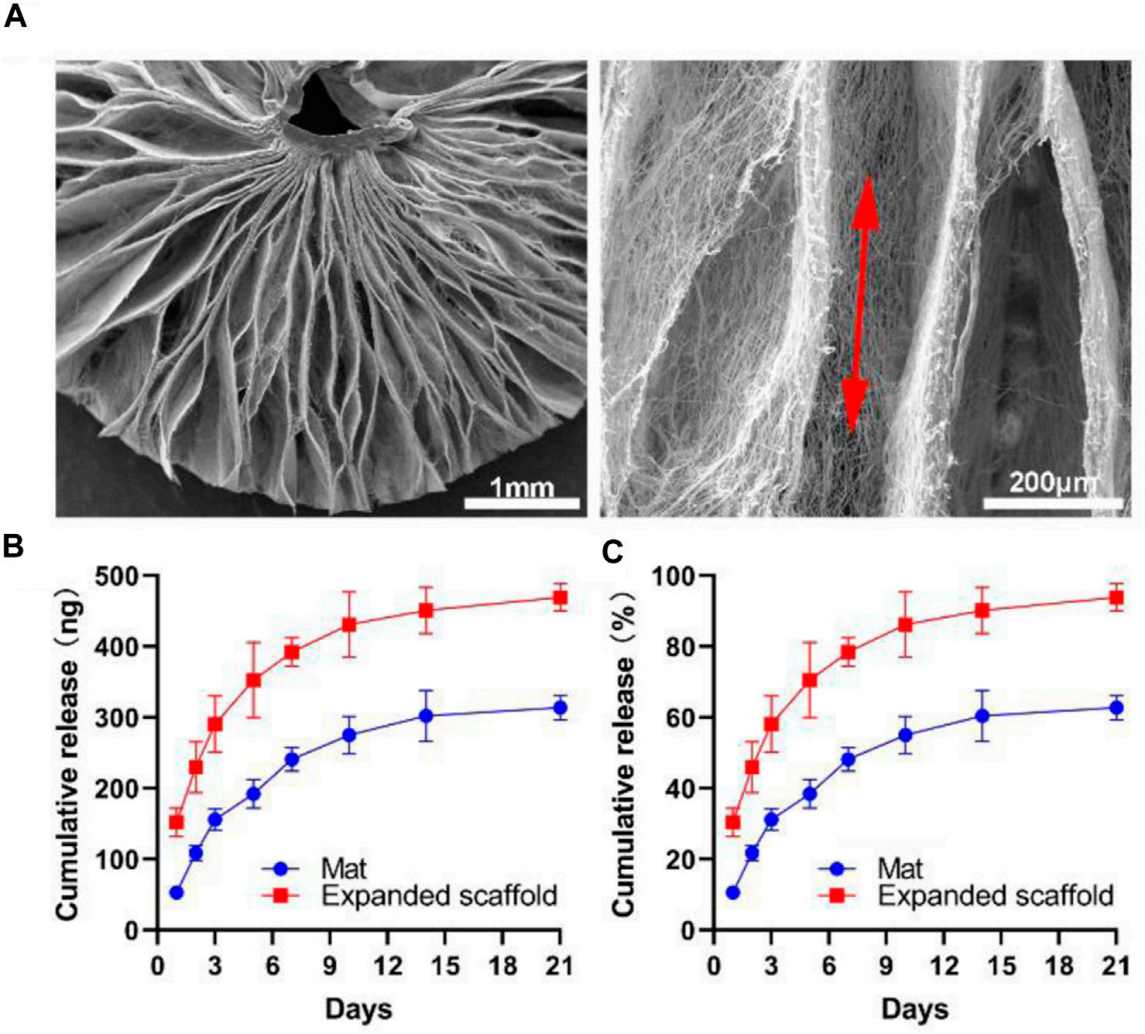
**(A)** SEM images show the structure, and fiber alignment of the 3D radially aligned PCL nanofiber scaffold expanded by depressurization of subcritical CO_2_ fluid. **(B, C)** *In vitro* release profiles of GM-CSF from PCL nanofiber mat and expanded nanofiber scaffold.

Figure 2A shows SEM images of a GM-CSF loaded, expanded, radially aligned PCL nanofiber scaffold by depressurization of subcritical CO_2_ fluid, discovering the scaffold consists of radially aligned nanofibers and gaps between neighboring nanofiber layers. Figures 2B, C show the release profiles of GM-CSF-loaded expanded radially aligned PCL nanofiber scaffolds and unexpanded nanofiber mats. An initial burst followed by a sustained release was exhibited over 3 weeks, and more encapsulated GM-CSF were released in the expanded radially aligned nanofiber scaffolds. The percentages of GM-CSF cumulative release after 3 weeks were (93.14 ± 3.61) % and (60.93 ± 2.81) % for GM-CSF-loaded PCL nanofiber mats before and after expansion, respectively. The presented GM-CSF release profile is similar to some excellent research by others (Ali et al., 2013; Ali et al., 2014; Bencherif et al., 2015; Verbeke and Mooney, 2015) which can ensure continuous recruitment of dendritic cells.

### 3D radially aligned nanofiber scaffolds guide cell migration and extracellular matrix deposition

**FIGURE 3:**
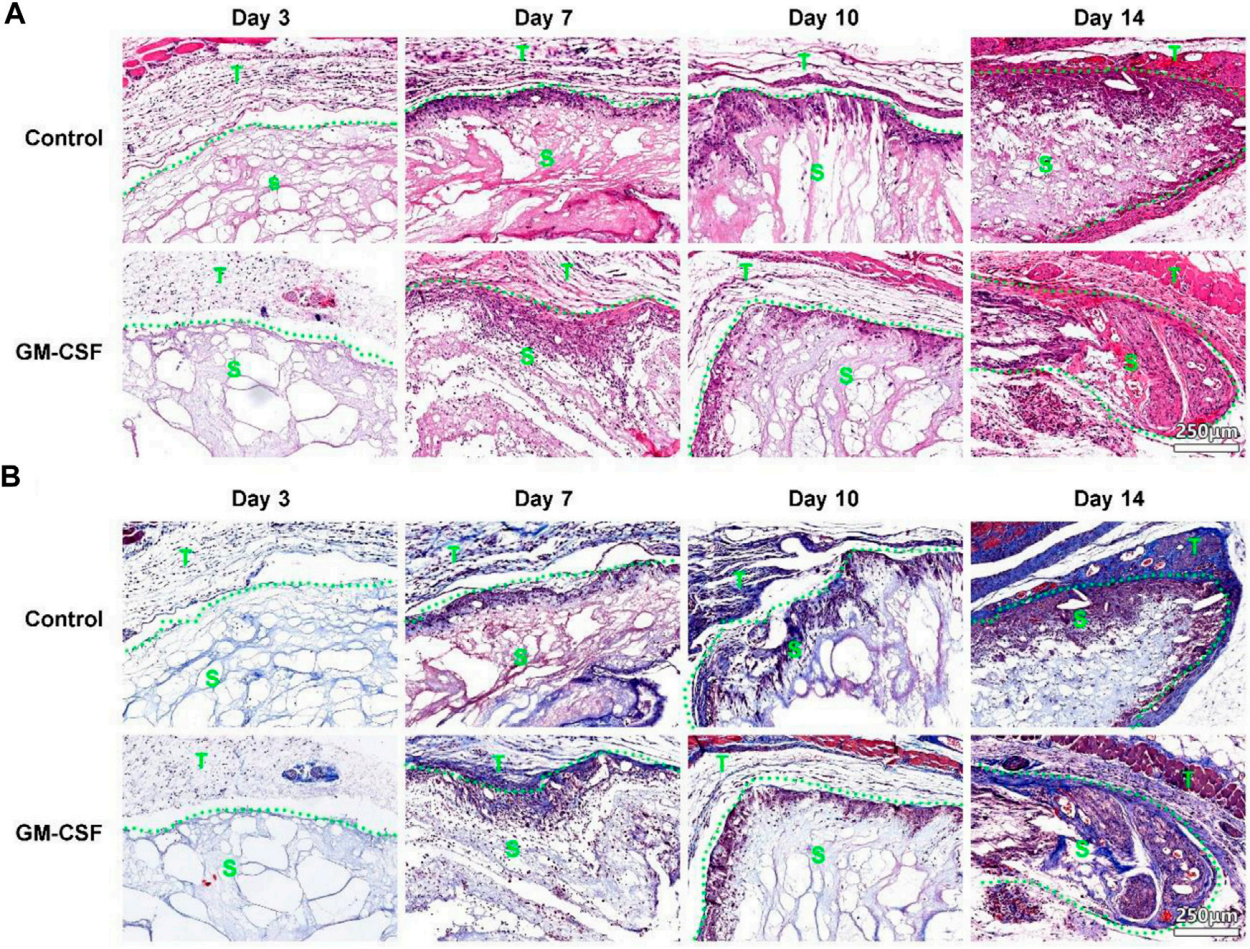
H&E **(A)** and Masson trichrome **(B)** staining of harvested expanded, radially aligned PCL nanofiber scaffolds without and with GM-CSF loading and surrounding tissues after subcutaneous implantation for 3, 7, 10, and 14 days. S: scaffold area (within the dotted line), T: tissue area (outside the dotted line).

After subcutaneous implantation, the H&E staining shows an increased cell penetration in both expanded radially aligned PCL nanofiber scaffolds with or without GM-CSF loading from day 3 to day 14. Cells infiltrated these scaffolds not only from the top and bottom surfaces but also from all sides (Figure 3A). Similarly, more and more collagen deposition was observed within the expanded radially aligned PCL nanofiber scaffolds with or without GM-CSF loading from day 3 to day 14. And there was no difference in cell penetration, and collagen deposition between the GM-CSF loaded scaffolds and no GM-CSF loaded scaffolds at all indicated time points (Figure 3B).

### GM-CSF could accelerate the expression of human dendritic cells (CD11c)

**FIGURE 4:**
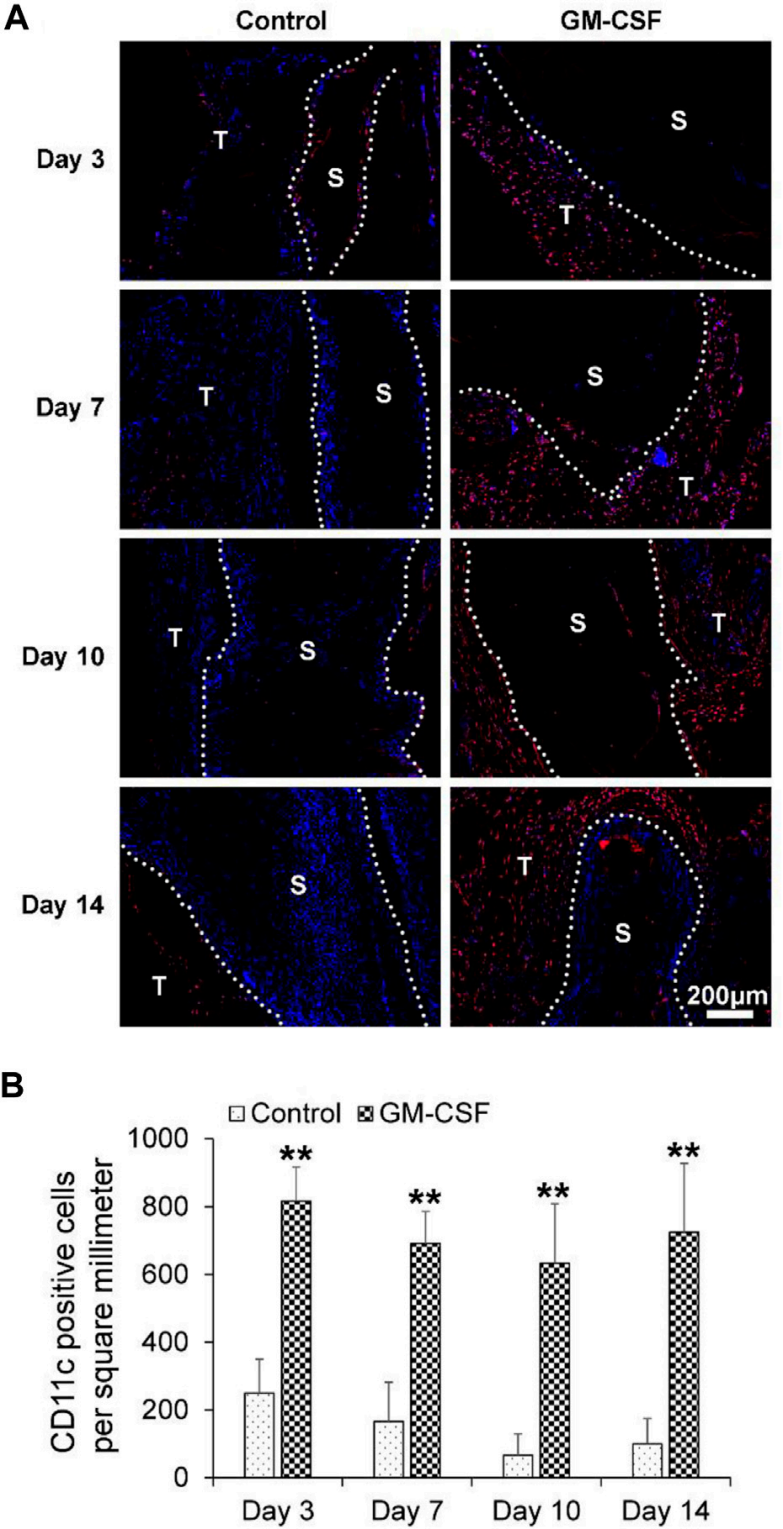
**(A)** The human dendritic cells (CD11c^+^) distribution in harvested expanded, radially aligned PCL nanofiber scaffolds without and with GM-CSF loading and their surrounding tissues after subcutaneous implantation for 3, 7, 10, and 14 days. S: scaffold area (within the dotted line), T: tissue area (outside the dotted line). **(B)** The quantification of CD11c^+^ positive cells within GM-CSF-loaded PCL nanofiber scaffolds area or PCL nanofiber scaffolds area after subcutaneous implantation for 3, 7, 10, and 14 weeks. ^**^ *p* < 0.01.

Here, we first examined the effects of GM-CSF on the recruitment of dendritic cells, which highly expressed CD11c. As shown in Figure 4, the expression of human CD11c within the expanded radially aligned PCL nanofiber scaffolds with GM-CSF loading was significantly higher than that within expanded radially aligned PCL nanofiber scaffolds after subcutaneous implantation for 3 (*p* < 0.01), 7 (*p* < 0.01), 10 (*p* < 0.01), and 14 (*p* < 0.01) days. These results indicate the locally released GM-CSF from implanted radially aligned nanofiber scaffold could enhance the expression of dendritic cells.

### GM-CSF could accelerate the expression of human cytotoxic T cells (CD3^+^/CD8^+^)

**FIGURE 5:**
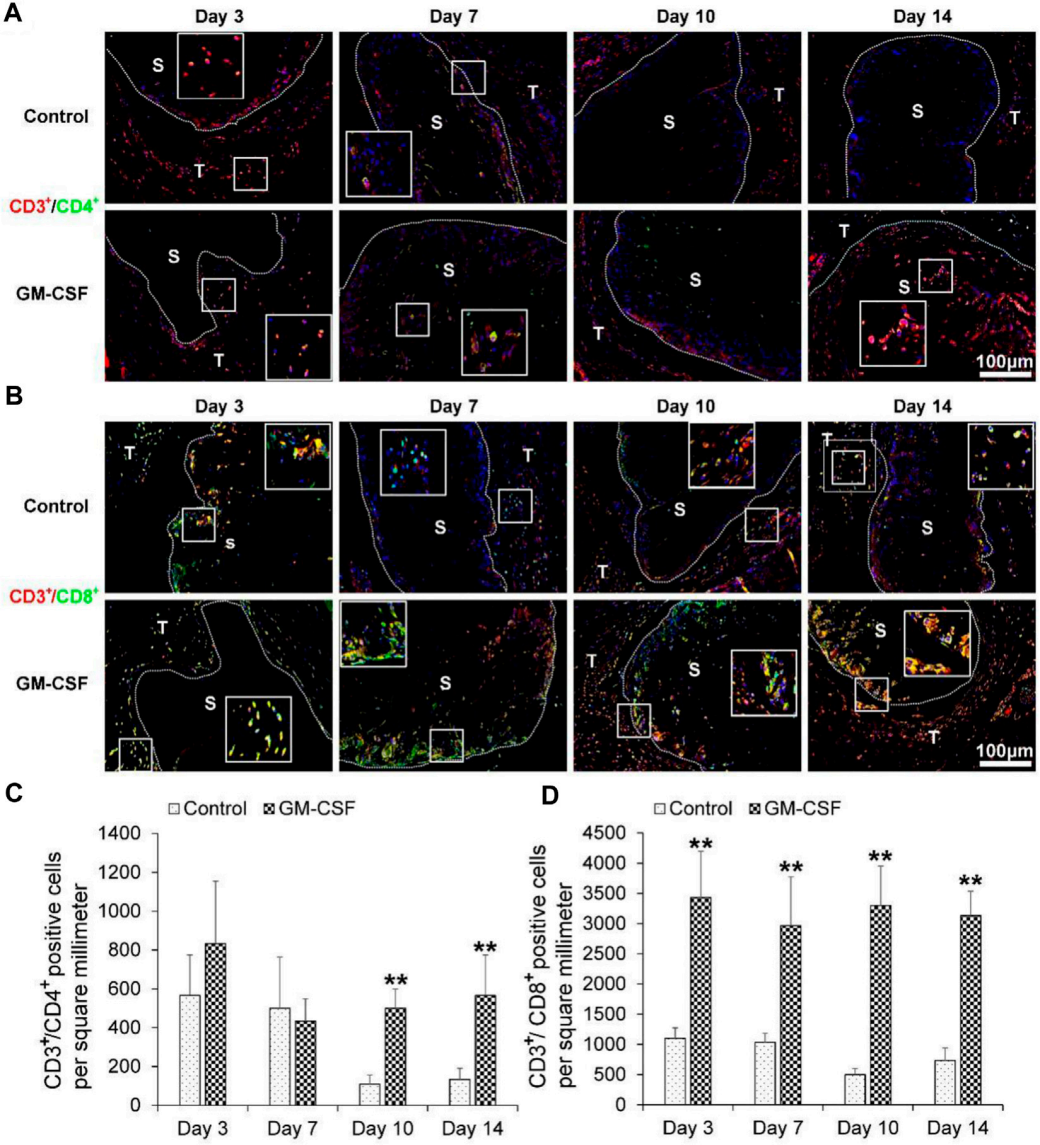
The human helper T cells (CD3^+^/CD4^+^) **(A)** and human cytotoxic T cells (CD3^+^/CD8^+^) **(B)** immunohistochemical staining of harvested expanded, radially aligned PCL nanofiber scaffolds without and with GM-CSF loading and their surrounding tissues after subcutaneous implantation for 3, 7, 10, and 14 days. S: scaffold area (within the dotted line), T; tissue area (outside the dotted line). The quantification of human CD3^+^/CD4^+^ **(C)** and human CD3^+^/CD8^+^ **(D)** positive cells within the GM-CSF-loaded PCL nanofiber scaffold area or PCL nanofiber scaffold area after subcutaneous implantation for 3, 7, 10, and 14 days. ^*^ *p* < 0.05, ^**^ *p* < 0.01.

Then we explored the expression of human helper T cells (CD3^+^/CD4^+^) (A) and human cytotoxic T cells (CD3^+^/CD8^+^). As shown in Figures 5A, C, there was no difference in the expression of human helper T cells (CD3^+^/CD4^+^) between the expanded radially aligned PCL nanofiber scaffolds with or without GM-CSF loading after subcutaneous implantation for 3 and 7 days. While the expression of human helper T cells (CD3^+^/CD4^+^) within expanded radially aligned PCL nanofiber scaffolds with GM-CSF loading was dramatically increased compared to that within expanded radially aligned PCL nanofiber scaffolds after 10 (*p* < 0.01) and 14 (*p* < 0.01) days of subcutaneous implantation. As shown in Figures 5B, D, the expression of human cytotoxic T cells (CD3^+^/CD8^+^) within expanded radially aligned PCL nanofiber scaffolds with GM-CSF loading was significantly increased compared to that within expanded radially aligned PCL nanofiber scaffolds after 3(*p* < 0.01), 7 (*p* < 0.01), 10 (*p* < 0.01) and 14 (*p* < 0.01) days of subcutaneous implantation. In other’s studies, the earliest detection point for dendritic cells and cytotoxic T cells is 5, 7, and 9 days after surgery, respectively (Ali et al., 2013; Bencherif et al., 2015; Verbeke and Mooney, 2015). While in our study, lots of dendritic cells and cytotoxic T cells were detected only 3 days after the operation. Suggesting the GM-CSF loaded radially aligned scaffolds can faster recruit dendritic cells and activate the cytotoxic T cells.

### GM-CSF could accelerate the expression of human M1 macrophages (CD68/CCR7)

**FIGURE 6:**
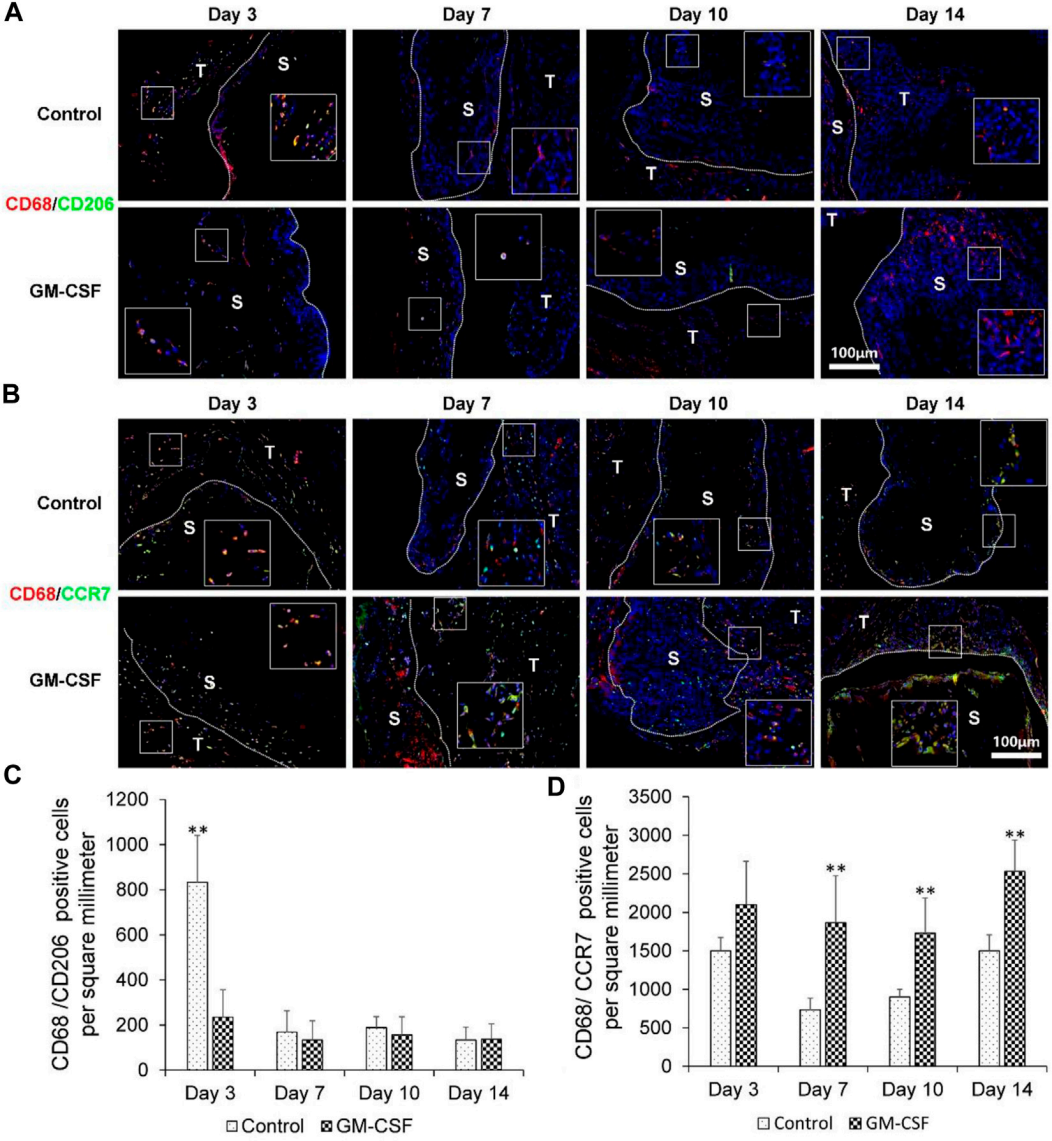
The human M2 macrophages (CD68/CD206) **(A)** and human M1 macrophages (CD68/CCR7) **(B)** immunohistochemical staining of harvested expanded, radially aligned PCL nanofiber scaffolds without and with GM-CSF loading and their surrounding tissues after subcutaneous implantation for 3, 7, 10, and 14 days. S: scaffold area (within the dotted line), T; tissue area (outside the dotted line). The quantification of human M2 macrophages (CD68/CD206) **(C)** and human M1 macrophages (CD68/CCR7) **(D)** positive cells within the GM-CSF-loaded PCL nanofiber scaffold area or PCL nanofiber scaffold area after subcutaneous implantation for 3, 7, 10, and 14 days. ^**^ *p* < 0.01.

Finally, we explored the expression of human M2 macrophages (CD68/CD206) and human M1 macrophages (CD68/CCR7). As shown in Figures 6A, C, the expression of M2 macrophages (CD68/CD206) of the control group was higher than the GM-CSF loaded 3D radially aligned nanofiber scaffold after subcutaneous implantation for 3 days. There was no difference in the expression of human M2 macrophages (CD68/CD206) between the expanded radially aligned PCL nanofiber scaffolds with or without GM-CSF loading after subcutaneous implantation for 7, 10, and 14 days. As shown in Figures 6B, D, there was no difference in the expression of human M1 macrophages (CD68/CCR7) between the 3D nanofiber scaffolds with or without GM-CSF loading after subcutaneous implantation for 3 days. However, the expression of human M1 macrophages (CD68/CCR7) of GM-CSF loaded nanofiber scaffold was significantly higher than the control group after subcutaneous implantation for 7, 10, and 14 days. Suggesting the GM-CSF loaded 3D radially aligned scaffolds also can activate the M1 macrophages.

## Discussion

Due to the high degradation rate of GM-CSF and its short half-life *in vivo* (Pettit et al., 1997; Groves and Schmidt-Lucke, 2000). Direct injection into the injury site does not provide the dwell time required for the growth factors to interact with the target cells in the wound area. Current growth factors loaded biomaterial scaffolds need higher doses or more frequent dosing, which may lead to unnecessary losses and side effects (Koria, 2012). Therefore, an effective delivery system is required to deliver growth factors to the injury site in a stable manner (Laiva et al., 2018). Nanotechnology has been applied to the scaffold preparation process to mimic the 3D porous topology of natural ECM. In previous studies, nanofibers are mostly randomly oriented, and the conventional 2D nanofiber membranes have limited their application to some extent due to the tight structure and small pore size. Our research focuses on preparing ordered PCL nanofiber mats loaded with GM-CSF in a radial pattern through electrostatic spinning technology. *In-situ* gas foaming of 2D nanofiber mats during the expansion process using the chemical foaming agent NaBH4 in our previous study (Chen et al., 2020a). The addition of surfactant (pluronic F-127) during the process causes the membrane to swell steadily and uniformly, thus transforming the 2D nanofiber mat into a 3D radially aligned radially shaped nanofiber scaffold. The final scaffold consists of radially aligned nanofibers and gaps between adjacent nanofiber layers. The nanoscale scaffolds synthesized in this study hold several characteristics: i) Nanofiber morphology mimics extracellular matrix structure, ii) The internal 3D radial arrangement structure, open pores provide enough space for cells, and the radial arrangement structure is more conducive to the migration and metabolism of cells. iii) By encapsulating growth factors and thus biochemical signals to direct cellular responses. GM-CSF was encapsulated in PCL nanofibers using co-axial electrospinning technology, and the final mid-release profile of the radially aligned nanofiber scaffolds loaded with GM-SCF was clarified by ELISA to release more GM-CSF due to the advantage of the internal pore size and porosity of the expanded scaffolds, thus ensuring the continuous recruitment of dendritic cells. One study used PLGA/PLA particles as a local delivery agent for GM-CSF, simulating the continuous release of GM-CSF in mice for at least 9 days, during which the continuous release of GM-CSF from the particle injection site resulted in local recruitment of neutrophils and macrophages (Pettit et al., 1997).

GM-CSF is a multifunctional growth factor primarily used in treating leukocyte deficiency and the immune deficiency caused by severe infection after radiation and chemotherapy for tumors (Hübel et al., 2002). The critical role of GM-CSF in wound healing is also receiving increasing attention. During wound healing, GM-CSF first diffuses into the surrounding tissues and chemically recruits neutrophils and monocytes into the wound, phagocytizing pathogens and secreting proteolytic enzymes (Min et al., 2018). Subsequently, cell proliferation and later collagen remodeling are promoted by modulating growth factors (Jorgensen et al., 2002). The inflammatory response is the earliest manifestation in wound healing. The locally released GM-CSF could enhance the recruitment of dendritic cells and the activation of cytotoxic T cells. Our study uses a humanized mouse subcutaneous implantation model to gain insight into the human immune response to radially aligned nanofiber scaffolds loaded with GM-CSF. The expression of dendritic cells (CD11c^+^), helper T cells (CD3^+^/CD4^+^), and cytotoxic T cells (CD3^+^/CD8^+^) was found to be significantly higher in the expanded radially aligned PCL nanofiber scaffold loaded with GM-CSF than it in the radially aligned nanofiber scaffold unloaded with GM-CSF. It has been shown that CD11c^+^ has a synergistic stimulatory effect on immune cells and is an essential regulator of CD4^+^ T cells and CD8^+^ T cells proliferation and function (Bachem et al., 2010). CD4^+^ T cells and CD8^+^ T cells are significantly present during skin wound healing and express regulatory or inflammatory cytokines within the wound (Lin et al., 2014). Our study found that significant cell migration and extracellular matrix deposition were observed within the expanded radially aligned nanofiber scaffolds with or without GM-CSF loading. This indicates no difference in cell infiltration and collagen deposition between scaffolds loaded with GM-CSF and scaffolds without GM-CSF loading. This suggests that GM-CSF may not affect normal body fibroblasts’ ability to synthesize collagen. It has been shown that GM-CSF promotes the average deposition of repaired collagen fibers in the defect by regulating TGF-β expression (Masucci, 1996). And we have previously demonstrated that 3D scaffolds composed of radially aligned nanofibers guide and facilitate the migration of bone marrow stem cells from the periphery to the middle (Chen et al., 2021). However, the role of GM-CSF in regulating the tissue remodeling phase needs to be further investigated.

## Conclusion

In summary, we developed GM-CSF loaded 3D radially aligned nanofiber scaffold to achieve immunoregulation and tissue regeneration at the same time. In this study, we used a humanized mice model to evaluate cell infiltration, granulation tissue formation, and local immune responses, which can mimic the actual situation in the human body. The 3D radially aligned structure could promote cell migration towards the center of the scaffold from the surrounding tissues, boosting wound healing. The local sustained release of GM-CSF can recruit human dendritic cells and activate human cytotoxic T cells and M1 macrophages. It helps remove necrotic tissue from the wound. The presented strategy shows excellent potential for wound healing of severe burns and chronic wounds. In our future study, for one thing, the activating factor of dendritic cells, cytosine-phosphodiesterguanine oligodeoxynucleotide (CpG ODN) will also be encapsulated into the 3D radially aligned scaffolds along with the GM-CSF. The maturation of dendritic cells will be further explored (Bencherif et al., 2015; Koshy and Mooney, 2016). For another, a wound model will be established in the humanized mice, and the effects of GM-CSF and CpG ODN co-loaded radially aligned scaffolds on wound healing will be explored.

## Data Availability

The raw data supporting the conclusion of this article will be made available by the authors, without undue reservation.
